# Hypoxia significantly reduces aminolaevulinic acid-induced protoporphyrin IX synthesis in EMT6 cells

**DOI:** 10.1038/sj.bjc.6690220

**Published:** 1999-03

**Authors:** I Georgakoudi, P C Keng, T H Foster

**Affiliations:** Department of Biochemistry and Biophysics, University of Rochester School of Medicine and Dentistry, 601 Elmwood Avenue, Rochester, NY, 14642, USA; University of Rochester Cancer Center, University of Rochester School of Medicine and Dentistry, 601 Elmwood Avenue, Rochester, NY, 14642, USA; Department of Radiology, University of Rochester School of Medicine and Dentistry, 601 Elmwood Avenue, Rochester, NY, 14642, USA; Department of Physics and Astronomy, University of Rochester, Rochester, NY, 14627, USA

**Keywords:** protoporphyrin IX, aminolaevulinic acid, hypoxia, cell density, proliferation rate

## Abstract

We have studied the effects of hypoxia on aminolaevulinic acid (ALA)-induced protoporphyrin IX (PpIX) synthesis in EMT6 monolayer cultures characterized by different cell densities and proliferation rates. Specifically, after ALA incubation under hypoxic or normoxic conditions, we detected spectrofluorometrically the PpIX content of the following populations: (a) low-density exponentially growing cells; (b) high-density fed-plateau cells; and (c) high-density unfed-plateau cells. These populations were selected either for the purpose of comparison with other in vitro studies (low-density exponentially growing cells) or as representatives of tumour regions adjacent to (high-density fed-plateau cells) and further away from (high-density unfed-plateau cells) capillaries. The amount of PpIX per cell produced by each one of these populations was higher after normoxic ALA incubation. The magnitude of the effect of hypoxia on PpIX synthesis was dependent on cell density and proliferation rate. A 42-fold decrease in PpIX fluorescence was observed for the high-density unfed-plateau cells. PpIX production by the low-density exponential cells was affected the least by ALA incubation under hypoxic conditions (1.4-fold decrease), whereas the effect on the high-density fed-plateau population was intermediate (20-fold decrease). © 1999 Cancer Research Campaign


*British Journal of Cancer* (1999) **79**(9/10), 1372–1377
© 1999 Cancer Research Campaign
Article no. bjoc.1998.0220

### Hypoxia significantly reduces aminolaevulinic acid-induced protoporphyrin IX synthesis in EMT6 cells

**I Georgakoudi[1], PC Keng[1,2] and TH Foster[1,2,3,4]**

Department of [1]Biochemistry and Biophysics, [2]University of Rochester Cancer Center and [3]Department of Radiology, University of Rochester School of Medicine and Dentistry, 601 Elmwood Avenue, Rochester, NY 14642; [4]Department of Physics and Astronomy, University of Rochester, Rochester, NY 14627, USA

**Summary** We have studied the effects of hypoxia on aminolaevulinic acid (ALA)-induced protoporphyrin IX (PpIX) synthesis in EMT6 monolayer cultures characterized by different cell densities and proliferation rates. Specifically, after ALA incubation under hypoxic or normoxic conditions, we detected spectrofluorometrically the PpIX content of the following populations: (a) low-density exponentially growing cells; (b) high-density fed-plateau cells; and (c) high-density unfed-plateau cells. These populations were selected either for the purpose of comparison with other in vitro studies (low-density exponentially growing cells) or as representatives of tumour regions adjacent to (high-density fed-plateau cells) and further away from (high-density unfed-plateau cells) capillaries. The amount of PpIX per cell produced by each one of these populations was higher after normoxic ALA incubation. The magnitude of the effect of hypoxia on PpIX synthesis was dependent on cell density and proliferation rate. A 42-fold decrease in PpIX fluorescence was observed for the high-density unfed-plateau cells. PpIX production by the low-density exponential cells was affected the least by ALA incubation under hypoxic conditions (1.4-fold decrease), whereas the effect on the high-density fed-plateau population was intermediate (20-fold decrease).

**Keywords:** protoporphyrin IX; aminolaevulinic acid; hypoxia; cell density; proliferation rate

Photodynamic therapy (PDT) of cancer combines the use of photosensitive drugs that accumulate preferentially in the tumour and irradiation with visible or near-infrared light. Cytotoxic reactions follow the optical excitation of the sensitizer to its triplet excited state that eventually lead to tumour eradication. Although most PDT photosensitizers are exogenously administered drugs, protoporphyrin IX (PpIX) is an effective endogenous sensitizer (Kennedy et al, 1990). PpIX is one of the intermediates in the haem biosynthetic pathway, and it has been shown that administration of excess aminolaevulinic acid (ALA) overrides one of the rate-limiting steps in this pathway and leads to accumulation of PpIX in the cells (Kennedy and Pottier, 1992; Peng et al, 1997). For reasons that are not yet completely understood, PpIX is often produced in higher amounts by malignant cells, conferring selective photodynamic damage to the tumour area. Although most researchers have taken advantage of the selective PpIX accumulation in the tumour to treat the malignancy by means of photodynamic therapy, others have used PpIX fluorescence detection as a tool for the diagnosis of disease (Kriegmair et al, 1994; Kennedy et al, 1996).

The effectiveness of PpIX as a PDT sensitizer and as a diagnostic agent has prompted a significant amount of research on the factors that affect PpIX synthesis. Such studies have explored the role of ALA incubation time (Dietel et al, 1996; Krammer and Uberriegler, 1996; Gibson et al, 1997) and concentration (Krammer and Uberriegler, 1996; Gibson et al, 1997; Moan et al, 1998), cell type (Iinuma et al, 1994; Steinback et al, 1995; Wyld et al, 1997), cell cycle (Moan et al, 1998), proliferation status (Rebeiz et al, 1992; Schick et al, 1995; Momma et al, 1997), serum presence (Hanania and Malik, 1992; Fukuda et al, 1993), iron availability (Hanania and Malik, 1992; Iinuma et al, 1994; Rittenhouse-Diakun et al, 1995; Tan et al, 1997), pH (Bermudez Moretti et al, 1993; Krammer and Uberriegler, 1996; Bech et al, 1997; Wyld et al, 1998), glucose (Dietel et al, 1996), and temperature (Dietel et al, 1996). More detailed studies examined the effect of some of these factors on specific enzymes of the haem biosynthetic pathway (Schoenfeld et al, 1988; Gibson et al, 1998).

The main aim of our study was to investigate the role of hypoxia in PpIX production by EMT6 cells. It has been shown that the mean oxygen tension of several tumours is lower than that of the corresponding normal tissue (Vaupel et al, 1989). In addition, the variability of oxygen tension within several types of tumours is well documented (Vaupel et al, 1989). Therefore, if oxygen availability affects PpIX synthesis, tumour heterogeneities in oxygen tension could result in corresponding differences in PpIX concentration and in the efficacy of tumour treatment or detection. A recent study by Wyld et al (1998) examined the effects of hypoxia on PpIX production by exponentially growing bladder cancer cells. In an attempt to simulate some of the different growth states of cells present in a tumour, we studied the effects of hypoxia on PpIX production for three cell populations: (a) low-density exponentially growing cells, included for comparison with other in vitro studies; (b) high-density fed-plateau cells, representing populations in close proximity to capillaries; and (c) high-density unfed-plateau cells, simulating the quiescent populations present in regions further away from capillaries. We find that ALA

*Received 29 April 1998*
*Revised 24 July 1998*
*Accepted 4 August 1998*

*Correspondence to:* TH Foster, Department of Radiology incubation of cells under hypoxia results in a significant decrease in PpIX synthesis, irrespective of cell density or growth phase. However, the magnitude of the effect is not the same for all populations. Specifically, the high-density unfed-plateau cells experience the largest decrease in PpIX production, followed by the high-density fed-plateau cells, which in turn are affected more than the low-density exponential population.

### MATERIALS AND METHODS

### Chemicals and reagents

Aminolaevulinic acid hydrochloride was purchased from Porphyrin Products (Logan, UT, USA). Solutions of 1 mM ALA were prepared fresh for each experiment by dissolving 8.7 mg ALA in 50 ml sterile Eagle's basal medium (BME). Cell culture media and antibiotics were purchased from Gibco (Grand Island, NY, USA); fetal bovine serum (FBS) was purchased from Atlanta Biologicals (Atlanta, GA, USA); glucose oxidase and RNase were purchased from Sigma Chemical (St. Louis, MO, USA); and propidium iodide was purchased from Molecular Probes (Eugene, OR, USA).

### Cell maintenance and culture

EMT6 cells were maintained in passage culture in 100 mm-diameter polystyrene dishes (Becton Dickinson, Franklin Lakes, NJ, USA) in 10 ml BME supplemented with 10% FBS, 50 units $ml^{-1}$ penicillin g, 50 µg $ml^{-1}$ streptomycin and 1.0 mg $ml^{-1}$ Fungizone (complete BME). Cultures were maintained at 37°C in a 95% air–5% carbon dioxide humidified atmosphere. Three different cell populations were used: (a) low-density exponentially growing cells; (b) high-density fed-plateau cells; and (c) high-density unfed-plateau cells. To establish the low-density exponential cells, $3 \times 10^5$ cells from passage culture were seeded on 100 mm-diameter polystyrene culture dishes. Two days later, $1 \times 10^5$ cells from the subsequent population were transferred to 60 mm glass Pyrex brand Petri dishes (VWR Scientific Products, Rochester, NY, USA) containing 5 ml complete BME. Glass dishes ensured that oxygen could not diffuse into the cells from the plate walls when cells were incubated under hypoxic conditions. The experiment was performed 2 days after the final seeding, and the medium was replaced once for these cells on the intervening day. The high-density populations were established by seeding $3.6 \times 10^6$ cells from passage culture on 60 mm glass Petri dishes containing 5 ml complete BME 7 days before the experiment. For the fed-plateau population, the medium was changed for the first time 3 days after the seeding and every subsequent day until the experiment. The medium of the high-density unfed-plateau cells was changed only once 3 days after the seeding.

### DNA staining assay

The assay performed to determine the growth phase of the cell populations has been described previously (Tsai, 1996). Briefly, on the day of the experiment two or four dishes were selected randomly from each one of the cell populations studied. The cells were removed from the plates by trypsinization (0.25% lyophilized trypsin in sterile distilled water), transferred to a 15 ml tube and centrifuged for 5 min at 1000 r.p.m. The supernatant was aspirated and the pellet was dissolved in 1 ml complete BME. A 30 µl sample was used for cell counting with a particle counter (Model ZM, Coulter Electronics, Hialeah, FL, USA). Two million cells were fixed in 6 ml 75% ethyl alcohol and stored at 4°C for at least 24 h. After fixation, the cells were treated with 1 ml RNase (1 mg $ml^{-1}$ in 1 × phosphate-buffered saline) for 30 min at room temperature and centrifuged for 7 min at 1000 r.p.m. After the addition of 750 µl propidium iodide (10 µg $ml^{-1}$ in 1 × phosphate-buffered saline), each sample was filtered through a 37 µm mesh and its cellular DNA content was analysed using an Epics Profile flow cytometer (Coulter Electronics). The percentage of cells in each of the cell cycle phases was determined by using the Multicycle AV commercial software package (Phoenix Flow System, San Diego, CA, USA).

### ALA administration under hypoxic conditions

Initially, the media of all samples were replaced by 3 ml fresh BME. We decreased the oxygen tension in the medium by introducing 270 µl glucose oxidase (20 units $ml^{-1}$ dissolved in distilled water) and placing the dishes in an evacuation chamber that was sparged every 20 min with 95% nitrogen–5% carbon dioxide. The chamber was in a warm room with the temperature maintained at 37°C. The oxygen tension, monitored for one sample with an oxygen sensitive microelectrode (Microelectrodes, Londonderry, NH, USA) calibrated in BME and BME with glucose oxidase, decreased to a value less than 0.4% within 40 min of incubation in the chamber. After exposure to hypoxia for 1 h, the evacuation chamber was opened, and the medium of the samples was replaced by either 3 ml fresh BME and 270 µl glucose oxidase (controls) or by 3 ml fresh BME containing 1 mM ALA and 270 µl glucose oxidase. The plates were placed again in the evacuation chamber and intermittent sparging with 95% nitrogen–5% carbon dioxide was performed every 20 min for 2 h. After a total ALA incubation time of 4 h in the dark, the cells were removed from the evacuation chamber and treated in the same manner as those sensitized with ALA under normoxic conditions.

### ALA administration under normoxic conditions

To maintain consistency with the protocol followed for ALA administration under hypoxic conditions, the samples' media were replaced initially by 3 ml BME. After 1 h, they were replaced again by either 3 ml BME (controls) or 3 ml BME containing 1 mM ALA and 270 µl BME without ALA to ensure that the cells were treated with equal concentrations of ALA under normoxic and hypoxic conditions. These samples were incubated in a 95% air–5% carbon dioxide atmosphere at 37°C for 4 h in the dark.

### Quantification of cellular PpIX content

After incubation either in BME or in ALA, each sample was washed once with 1 ml minimal essential medium without phenol red (MEM) and treated with 0.5 ml 0.25% trypsin until the cells started detaching from the plates. The trypsinization process was halted by the addition of 2.5 ml MEM containing 10% FBS and the cells were transferred to a 15 ml tube. After centrifugation for 5 min at 1 000 r.p.m., the supernatant was aspirated and different amounts of MEM were added to the low-density exponential (0.5 ml), high-density fed-plateau (4.5 ml), and high-density unfed-plateau (3.5 ml) cell populations so that the resulting cell

© Cancer Research Campaign 1999

suspensions would have approximately equal cellular concentrations (about $7.5 \times 10^5$ cells $ml^{-1}$). A 100 μl sample from each tube was diluted in 20 ml saline and used to count the exact number of cells corresponding to each one of the dishes that were treated with BME or ALA. Three hundred and fifty microlitres from each cell sample were pipetted into a microcell (Spex Industries, Edison, NJ, USA) and the front face fluorescence spectrum was recorded by a spectrofluorometer (Fluorolog 2, SPEX Industries). The excitation wavelength was set at 405 nm, the integration time was 0.5 s, the resolution was 1 nm and fluorescence was detected from 550 to 730 nm. It should be noted that after trypsinization the cell samples remained in ice during the time they were not handled. The PpIX content of the incubation medium was not measured because it did not contain serum; a previous study has shown that only a minimal amount of PpIX leaks out of the cells when ALA incubation occurs in the absence of serum (Hanania and Malik, 1992).

Individual experiments were performed either with low-density exponential and high-density unfed-plateau cells or with high-density fed-plateau cells. Half of the samples from each population were treated under normoxic conditions, whereas the other half were treated under hypoxic conditions. For each of the three experiments with the low-density exponential and high-density unfed-plateau cells, we had four dishes for each treatment group: two of them served as controls (incubated with BME) and the other two were incubated with ALA. During the experiment performed with the high-density fed-plateau exponential cells, eight dishes were incubated under normoxic conditions and eight were incubated under hypoxic conditions. Two samples in each group were treated with BME (controls), whereas the other six were treated with ALA.

### Statistical analysis

The equal tails Student's *t*-test (Crow et al, 1960) was used to determine statistical differences in the mean values of the normalized peak PpIX fluorescence signal (634 nm) corresponding to each one of the cell populations studied.

### RESULTS

Shown in Figure 1 are representative DNA content histograms obtained from flow cytometric analysis of propidium iodide-stained cells from the three cell populations considered. The percentage of cells in the S-phase of the cell cycle, i.e. the phase during which DNA is synthesized, was used as an indicator of the proliferating status of the population. These percentages are 42.15 ± 6.7 ($n$ = 6), 34.9 ± 2.4 ($n$ = 4) and 11.9 ± 2.3 ($n$ = 6) for the low-density exponential (Figure 1A), high-density fed-plateau (Figure 1B), and high-density unfed-plateau cells (Figure 1C), respectively (mean ± standard deviation reported).

PpIX spectra corresponding to the three cell populations treated with 1 mM ALA for 4 h under normoxic (Figure 2A) and hypoxic (Figure 2B) conditions are displayed in Figure 2. The background fluorescence, i.e. the fluorescence not associated with ALA-induced fluorophores, is considered to be the same as the spectrum obtained from the control samples. Thus, we subtract the spectrum of a control sample from the spectrum of the corresponding ALA-treated group and divide it by the number of cells in the sample to obtain each one of the spectra shown in Figure 2. We should note that the autofluorescence spectra from all of the

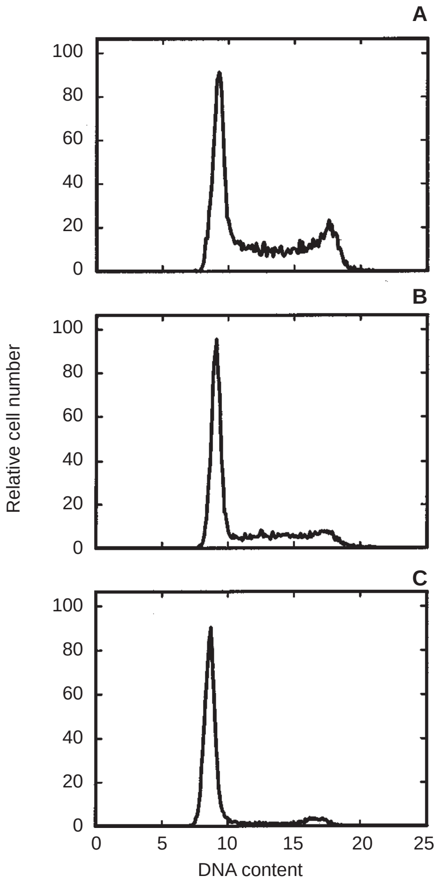

**Figure 1** Representative DNA content histograms obtained by flow cytometric analysis of propidium iodide stained cells from: (**A**) the low-density exponential population; (**B**) the high-density fed-plateau population; and (**C**) the high-density unfed-plateau population. The percentage of cells in the S-phase as determined by a curve fitting algorithm is 42.15 ± 6.7, 34.9 ± 2.4 and 11.9 ± 2.3 for (**A**), (**B**) and (**C**) respectively (mean ± standard deviation from at least four different measurements is reported in each case). The coefficient of variance for the G1 peaks was less than 5% for all cases control samples were very similar, regardless of incubation conditions, cell density or proliferation rate (data not shown). The characteristic PpIX peak at approximately 634 nm is present in all spectra, whereas the lower intensity peak at 705 nm is not clearly visible for the high-density unfed-plateau cells treated with ALA under hypoxia because of the low overall PpIX fluorescence intensity detected in this case. We observe that the intensity of PpIX fluorescence is dependent on cell density and proliferation rate. More importantly, we find that hypoxia affects PpIX production in very significant ways.

These trends are displayed in a more quantitative manner in Figure 3. For each experiment, the background-subtracted peak PpIX fluorescence per cell from each sample is normalized to that of one of the low-density exponential populations treated with ALA under normoxic conditions. Thus, the normalized peak PpIX fluorescence per cell of the normoxic low-density exponential group approaches unity. When cells are treated with ALA under normoxic conditions, we find that the normalized peak PpIX fluorescence of the high-density fed-plateau population (3.1 ± 0.4) is not statistically different from that of the high-density unfed-plateau population (2.5 ± 0.9) for $P < 0.05$. The peak PpIX fluorescence of both high-density plateau populations is higher than

 © Cancer Research Campaign 1999

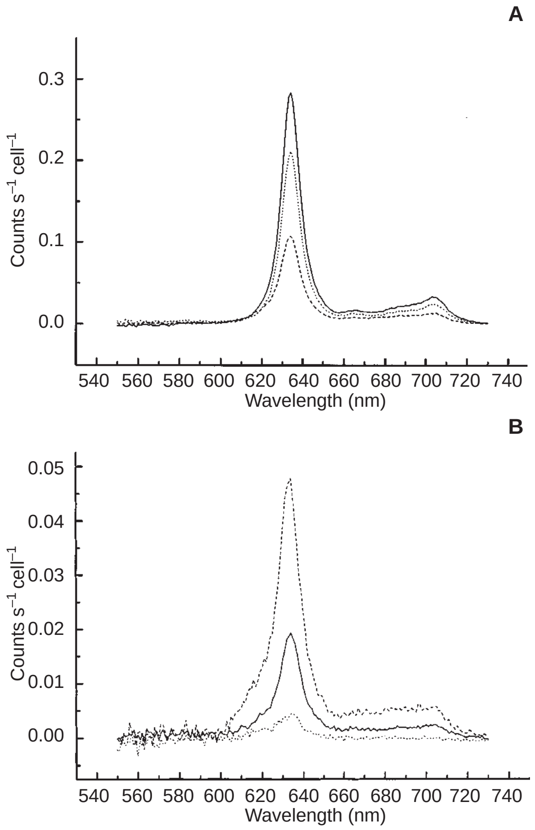

**Figure 2** Representative background subtracted PpIX spectra obtained from whole cell samples treated with 1 mM ALA for 4 h under (**A**) normoxic or (**B**) hypoxic conditions. Spectra from the high-density fed-plateau (——), high-density unfed-plateau (……) and low-density exponential (- - - - -) populations are shown in **A** and **B**. The characteristic PpIX peak at 634 nm is present in all spectra, but the 705-nm peak is not detectable for the hypoxic high-density unfed-plateau population because of the overall decrease in the fluorescence

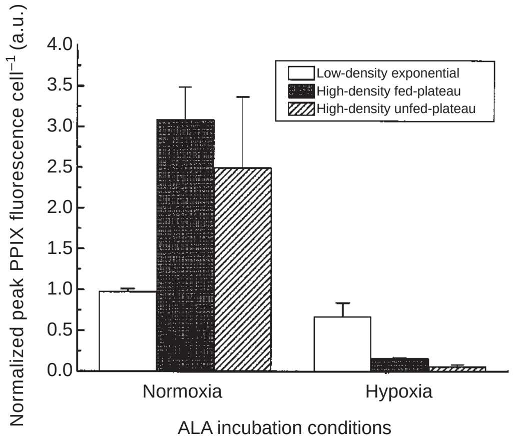

**Figure 3** Hypoxia significantly affects PpIX synthesis for all populations considered. The magnitude of this effect is dependent on cell density and proliferation rate. The normalized peak PpIX fluorescence cell$^{-1}$ is 2.5 ± 0.9, 3.1 ± 0.4 and 0.98 ± 0.03 for the normoxic high-density unfed-plateau, high-density fed-plateau, and low-density exponential cells respectively. The normalized peak PpIX fluorescence for these populations, in the same order, treated with ALA under hypoxic conditions is 0.06 ± 0.02, 0.16 ± 0.015 and 0.7 ± 0.2 respectively (mean ± standard deviations reported; *n* = 6)

that of the low-density exponential group (0.98 ± 0.03; $P < 0.01$). However, when cells are treated with ALA under hypoxia, we find that the normalized peak PpIX fluorescence per cell produced by the low-density exponential cells (0.7 ± 0.2) is statistically higher than that of the high-density fed-plateau population (0.16 ± 0.015; $P < 0.01$), which in turn is higher than that of the high-density unfed-plateau group (0.06 ± 0.02; $P < 0.01$) (in each case, the mean ± standard deviation is reported; $n = 6$). The units reported are arbitrary because the purpose of this study was to examine in a qualitative manner the effects of hypoxia. Therefore, a calibration curve that could possibly allow us to quantitate the absolute intracellular PpIX concentrations was not obtained.

### DISCUSSION

The main purpose of this study was to determine the effects of hypoxia on the synthesis of PpIX by three cell populations that are characterized by different cell densities and proliferation rates. Our work with EMT6 monolayer cultures demonstrates that oxygen deprivation has a significant effect on PpIX production, the magnitude of which is dependent on the level of cell confluency and the proliferation status of the population.

Our whole cell fluorescence spectroscopy studies indicate that under normoxic ALA incubation conditions cell density is a factor that affects PpIX synthesis. Specifically, we find that the high-density populations produce substantially higher amounts of PpIX per cell than the low-density population. These results agree with fluorescence microscopy and flow cytometry studies performed by Moan et al (1998) with human colon adenocarcinoma cells and Chinese hamster lung fibroblasts. Steinbach et al (1995) observed an increasing amount of PpIX fluorescence with increasing cell density for two malignant and a normal urothelial cell line. However, cell density had no effect on PpIX production by N1 normal fibroblasts. Moreover, cell confluency was not an important determinant of PpIX production in human skin fibroblasts (Krammer and Uberriegler, 1996). Therefore, it appears that the effects of cell density on PpIX production could be cell line dependent. However, it is interesting to note that all of the malignant cell lines studied exhibit a similar cell confluency dependence.

When we compare PpIX synthesis by the two high-density populations under normoxic ALA incubation conditions, we find that there are not any significant differences between the fed-plateau and the unfed-plateau cells, even though their corresponding proliferation rates are very different as indicated by the percentage of cells in the S-phase for each group. These results are in agreement with studies performed by Fukuda et al (1993) showing that there is little obvious cell cycle dependence on PpIX generation by CNCM-I-221 mammalian epithelial cells. Moan et al (1998) have reported that the only cell cycle dependence of PpIX synthesis appears to be a simple volume dependence, with cells in the G2+M phases producing 1.9 times more PpIX than cells in the G1 phase. The higher mean PpIX fluorescence for the fed-plateau population could be explained by cell size-dependent PpIX production; however, our experiments suggest that the differences in the number of cells corresponding to each cell cycle phase for the high-density fed-plateau and unfed-plateau cells are not significant enough to result in statistically different PpIX fluorescence measurements. Momma et al (1997) and Iinuma et al (1994) have found also that the PpIX content of several malignant cell lines did not correlate with their different growth rates. Wyld et al (1997) reported similar results with six different cell lines that included fibroblasts, smooth muscle, endothelial and malignant cells. However, they noted that the plateau populations of three non-malignant cell lines contained a reduced intracellular amount of PpIX compared with the exponential populations, whereas the reverse was true for the two malignant cell lines that were included. As the confluency levels were different for the exponential and plateau populations, it is not clear whether the observed differences are a result of variation in cell densities or cell proliferation rates. The effects of cell stimulation by different factors on PpIX synthesis have been investigated as well. Schick et al (1995) found that although stimulation of normal human keratinocytes with epidermal growth factor α (EGF-α), a cytokine stimulating the proliferation of epidermal and epithelial cells, resulted in higher cytotoxicities with ALA-PDT, activation of two skin squamous cell carcinoma lines did not have any effects. Rebeiz et al (1992) observed a significant increase in PpIX accumulation when resting splenocytes were activated with the mitogenic lectin concavalin A. A similar effect was reported by Rittenhouse-Diakun et al (1995) when human peripheral blood lymphocytes were stimulated with three different mitogens. Thus, it appears that cell proliferation rate could affect PpIX synthesis, depending on the cell line.

It is possible that the slight decrease in PpIX production by the unfed-plateau cells compared with that of the fed-plateau cells is because of small pH differences between the two populations induced by the different tissue culture protocols that were followed to establish them. However, we did not observe any significant colour changes of the cells' media at the time they were harvested for the experiment. Studies reported by Bech et al (1997) and Wyld et al (1998) demonstrating that pH can affect PpIX synthesis were performed with cells that were exposed to different pH values during incubation with ALA. As mentioned in the Materials and Methods section, in our experiments the cells were incubated with fresh media 1 h before and during ALA incubation. Therefore, we do not suspect that any pH differences resulting from our tissue culture methods had a significant effect on PpIX production during this study.

Both cell confluency and proliferation state are important determinants of the magnitude of the effect of hypoxic ALA incubation on PpIX synthesis. Specifically, we find that although PpIX production under hypoxic conditions decreases by a factor of 1.4 for low-density exponential cells, it decreases by approximately 20-fold for high-density fed-plateau cells and 42-fold for high-density unfed-plateau cells. It was shown by Falk et al (1959) that variations in oxygen tension significantly affect the production of haem, protoporphyrin and coproporphyrin in whole blood cells from normal chickens. Recently, Wyld et al (1998) reported that PpIX production by exponentially growing bladder cancer cells was reduced significantly when ALA incubation was performed at 0%, 2.5% and 5% oxygen conditions when compared with that at 21% oxygen. However, to our knowledge, the work presented in this report is the first systematic study of the effects of hypoxia on ALA-induced PpIX production by cells at different proliferating states. The mechanisms that lead to decreased PpIX synthesis under hypoxic ALA incubation conditions remain to be studied. It is possible that ALA uptake is affected. It has been reported by Bermudez-Moretti et al (1993) that an active mechanism is involved in ALA transport in *Saccharomyces cerevisiae*. However, no temperature dependence was found in [$^{14}$C]ALA uptake by malignant human mammary and mesothelioma cells or by rat mammary adenocarcinoma cells, suggesting that an energy-dependent carrier is not involved (Gibson et al, 1997). Hypoxia could lead to decreased synthesis or activity of the enzymes that are involved in the haem biosynthetic pathway. The absence of any detectable changes in the shape of the recorded fluorescence spectra prevents us from suggesting any enzymes that could be affected in particular by limited oxygen availability. However, Sano and Granick (1961) have reported that coproporphyrinogen oxidase requires oxygen to act upon coproporphyrinogen. Moreover, Poulson and Polglase (1975) and Poulson (1976) have shown that oxygen is essential for protoporphyrinogen oxidase activity, the enzyme catalysing the oxidation of protoporphyrinogen IX to protoporphyrin IX. If excess protoporphyrinogen IX produced as a result of decreased protoporphyrinogen oxidase activity leaked from the mitochondria into the cytosol, it could be converted to PpIX when the cells were reoxidized during preparation for the spectrofluorometry studies (Fingar et al, 1997). Thus, the actual decrease in PpIX synthesis under hypoxia could be even more dramatic than our current estimates. Changes in the cellular glucose content could also be a factor because glucose concentration has been reported by Dietel et al (1996) to affect PpIX synthesis. We should note that the addition of glucose oxidase in the medium to induce a rapid decrease in oxygen concentration should not affect the glucose concentration of the medium (5.5 mM) because only approximately 240 μM glucose would be consumed in the process.

It is not clear why different cell densities and proliferation rates result in different hypoxic effects on PpIX production. A cell density-dependent modulation of genes that encode rate-limiting enzymes such as porphobilinogen deaminase and ferrochelatase has been suggested by Moan et al (1998). This modulation could lead to increased PpIX synthesis with increasing cell density under normoxic ALA incubation conditions, but have the opposite effect under hypoxic conditions. It is possible that a decreased iron content in exponentially growing cells could explain why they produce more PpIX than the plateau cells under hypoxia because decreased cellular iron content has been correlated with increased PpIX accumulation (Hanania and Malik, 1992; Rittenhouse-Diakun et al, 1995; Tan et al, 1997). However, this hypothesis is not consistent with the level of PpIX synthesis that we observe for the different cell populations under normoxic ALA incubation conditions.

In conclusion, this report demonstrates that hypoxia significantly affects PpIX production in EMT6 monolayer cultures. The effects of hypoxia appear to be dependent on cell density and proliferation status. If the trends observed with our monolayer cultures are relevant to the in vivo situation, then our results would suggest that the efficacy of PpIX as a photodynamic and fluorescence detection agent might be compromised in tumour regions that are hypoxic. Such effects should be considered when designing efficient tumour treatment or diagnostic protocols.

### ACKNOWLEDGEMENTS

This work was supported by USPHS grants CA68409, CA36856 and CA11198. The authors wish to thank Dr John Ludlow and Dr Nancy Krucher for the use of their evacuation chambers and assistance with hypoxia induction; Dr Philip Fay for the loan of the microcell used to perform fluorescence measurements; Dr Russel

 © Cancer Research Campaign 1999

Hilf and Mr Scott Gibson for helpful discussions on PpIX synthesis; Mr Jim Havens for expert technical assistance in tissue culture maintenance; Ms Regina Harley for expert technical assistance with the flow cytometer measurements; Ms Mylien Nguyen for instruction in the use of the spectrofluorometer; and Ms Cathy Cox for instruction in the DNA-staining assay and assistance during the early part of this project. One of the authors (IG) was partially supported by the University of Rochester Messersmith Fellowship.

### REFERENCES

Bech Ø, Berg K and Moan J (1997) The pH dependency of protoporphyrin IX formation in cells incubated with 5-aminolevulinic acid. *Cancer Lett* **113**: 25–29

Bermúdez Moretti M, Correa García S, Stella C, Ramos E and Del C Batlle AMC (1993) δ-aminolevulinic acid transport in *Saccharomyces cerevisiae*. *Int J Biochem* **25**: 1917–1924

Crow EL, Davis FA and Maxfield MW (1960) *Statistics Manual*. Dover Publications: New York

Dietel W, Bolsen K, Dickson E, Fritsch C, Pottier R and Wendenburg R (1996) Formation of water-soluble porphyrins and protoporphyrin IX in 5-aminolevulinic-acid-incubated carcinoma cells. *J Photochem Photobiol B*: *Biol* **33**: 225–231

Falk JE, Porra RJ, Brown A, Moss F and Larminie HE (1959) Effect of oxygen tension on haem and porphyrin biosynthesis. *Nature* **184**: 1217–1219

Fingar VH, Wieman TJ, McMahon KS, Haydon PS, Halling BP, Yuhas DA and Winkelman JW (1997) Photodynamic therapy using a protoporphyrinogen oxidase inhibitor. *Cancer Res* **57**: 4551–4556

Fukuda H, Batlle AMC and Riley PA (1993) Kinetics of porphyrin accumulation in cultured epithelial cells exposed to ALA. *Int J Biochem* **25**: 1407–1410

Gibson SL, Havens JJ, Foster TH and Hilf R (1997) Time-dependent intracellular accumulation of δ-aminolevulinic acid, induction of porphyrin synthesis and subsequent phototoxicity. *Photochem Photobiol* **65**: 416–421

Gibson SL, Cupriks DJ, Havens JJ, Nguyen ML and Hilf R (1998) A regulatory role for porphobilinogen deaminase (PBGD) in δ-aminolaevulinic acid (δ-ALA)-induced photosensitization? *Br J Cancer* **77**: 235–243

Hanania J and Malik Z (1992) The effect of EDTA and serum on endogenous porphyrin accumulation and photodynamic sensitization of human K562 leukemic cells. *Cancer Lett* **65**: 127–131

Iinuma S, Farshi SS, Ortel B and Hasan T (1994) A mechanistic study of cellular photodestruction with 5-aminolevulinic acid-induced porphyrin. *Br J Cancer* **70**: 21–28

Kennedy JC and Pottier RH (1992) Endogenous protoporphyrin IX, a clinically useful photosensitizer for photodynamic therapy. *J Photochem Photobiol B*: *Biol* **14**: 275–292

Kennedy JC, Pottier RH and Pross DC (1990) Photodynamic therapy with endogenous protoporphyrin IX: basic principle and present clinical experience. *J Photochem Photobiol B*: *Biol* **6**: 143–148

Kennedy JC, Marcus SL and Pottier RH (1996) Photodynamic therapy (PDT) and photodiagnosis (PD) using endogenous photosensitization induced by 5-aminolevulinic acid (ALA): mechanisms and clinical results. *J. Clin Laser Med Surg* **14**: 289–304

Krammer B and Überriegler K (1996) In-vitro investigation of ALA-induced protoporphyrin IX. *J Photochem Photobiol B*: *Biol* **36**: 121–126

Kriegmair M, Baumgartner R and Knüchel R (1994) Fluorescence photodetection of neoplastic urothelial lesions following intravesical instillation of 5-aminolevulinic acid. *Urology* **44**: 836–841

Moan J, Bech Ø, Gaullier J-M, Stokke T, Steen HB, Ma LW and Berg K (1998) Protoporphyrin IX accumulation in cells treated with 5-aminolevulinic acid: dependence on cell density, cell size and cell cycle. *Int J Cancer* **75**: 134–139

Momma T, Hamblin MR and Hasan T (1997) Hormonal modulation of the accumulation of 5-aminolevulinic acid-induced protoporphyrin and phototoxicity in prostate cancer cells. *Int J Cancer* **72**: 1062–1069

Peng Q, Warloe T, Berg K, Moan J, Kongshaug M, Giecksky K-E and Nesland JM (1997) 5-Aminolevulinic acid-based photodynamic therapy. *Cancer* **79**: 2282–2308

Poulson R (1976) The enzymic conversion of protoporphyrinogen IX to protoporphyrin IX in mammalian mitochondria. *J Biol Chem* **251**: 3730–3733

Poulson R and Polglase WJ (1975) The enzymic conversion of protoporphyrinogen IX to protoporphyrin IX. *J Biol Chem* **250**: 1269–1274

Rebeiz N, Rebeiz CC, Arkins S, Kelley KW and Rebeiz CA (1992) Photodestruction of tumor cells by induction of endogenous accumulation of protoporphyrin IX: enhancement by 1,10-phenanthroline. *Photochem Photobiol* **55**: 431–435

Rittenhouse-Diakun K, van Leengoed H, Morgan J, Hryhorenko E, Paszkiewicz G, Whitaker JE and Oseroff AR (1995) The role of transferrin receptor (CD71) in photodynamic therapy of activated and malignant lymphocytes using the heme precursor δ-aminolevulinic acid (ALA). *Photochem Photobiol* **61**: 523–528

Sano S and Granick S (1961) Mitochondrial coproporphyrinogen oxidase and protoporphyrin formation. *J Biol Chem* **236**: 1173–1180

Schick E, Kaufmann R, Rück A, Hainzl A and Boehncke W-H (1995) Influence of activation and differentiation of cells on the effectiveness of photodynamic therapy. *Acta Derm Venereol* **75**: 276–279

Schoenfeld N, Epstein O, Lahav M, Mamer R, Shaklai M and Atsmon A (1988) The heme biosynthetic pathway in lymphocytes of patients with malignant lymphoproliferative disorders. *Cancer Lett* **43**: 43–48

Steinbach P, Weingandt H, Baumgartner R, Kriegmair M, Hofstädter F and Knüchel R (1995) Cellular fluorescence of the endogenous photosensitizer protoporphyrin IX following exposure to 5-aminolevulinic acid. *Photochem Photobiol* **62**: 887–895

Tan WC, Krasner N, O'Toole P and Lombard M (1997) Enhancement of photodynamic therapy in gastric cancer cells by removal of iron. *Gut* **41**: 14–18

Tsai MA, Waugh RE and Keng PC (1996) Cell cycle-dependence of HL-60 cell deformability. *Biophys J* **70**: 2023–2029

Vaupel P, Kallinowski F and Okunieff P (1989) Blood flow, oxygen and nutrient supply, and metabolic microenvironment of human tumors: a review. *Cancer Res* **49**: 6449–6465

Wyld L, Burn JL, Reed MWR and Brown NJ (1997) Factors affecting aminolaevulinic acid-induced generation of protoporphyrin IX. *Br J Cancer* **76**: 705–712

Wyld L, Reed MWR and Brown NJ (1998) The influence of hypoxia and pH on

*© Cancer Research Campaign 1999*
